# Pulmonary Cryptococcosis

**DOI:** 10.3390/jof8111156

**Published:** 2022-10-31

**Authors:** Annaleise R. Howard-Jones, Rebecca Sparks, David Pham, Catriona Halliday, Justin Beardsley, Sharon C.-A. Chen

**Affiliations:** 1 Centre for Infectious Diseases & Microbiology Laboratory Services, New South Wales Health Pathology—Institute of Clinical Pathology & Medical Research, Westmead Hospital, Westmead, NSW 2145, Australia; 2 Faculty of Medicine and Health, University of Sydney, Camperdown, NSW 2145, Australia; 3 Sydney Institute for Infectious Diseases, The University of Sydney, Sydney, NSW 2006, Australia; 4 Westmead Institute for Medical Research, Westmead, NSW 2145, Australia

**Keywords:** cryptococcosis, diagnosis, antifungal agents, immunosuppression

## Abstract

Pulmonary cryptococcosis describes an invasive lung mycosis caused by *Cryptococcus neoformans* or *Cryptococcus gattii* complex. It is often a high-consequence disease in both immunocompromised and immunocompetent populations, and may be misdiagnosed as pulmonary malignancy, leading to a delay in therapy. Epidemiology follows that of cryptococcal meningoencephalitis, with *C. gattii* infection more common in certain geographic regions. Diagnostic tools include histopathology, microscopy and culture, and the detection of cryptococcal polysaccharide antigen or *Cryptococcus*-derived nucleic acids. All patients with lung cryptococcosis should have a lumbar puncture and cerebral imaging to exclude central nervous system disease. Radiology is key, both as an adjunct to laboratory testing and as the initial means of detection in asymptomatic patients or those with non-specific symptoms. Pulmonary cryptococcomas (single or multiple) may also be associated with disseminated disease and/or cryptococcal meningitis, requiring prolonged treatment regimens. Optimal management for severe disease requires extended induction (amphotericin B and flucytosine) and consolidation therapy (fluconazole) with close clinical monitoring. Susceptibility testing is of value for epidemiology and in regions where relatively high minimum inhibitory concentrations to azoles (particularly fluconazole) have been noted. Novel diagnostic tools and therapeutic agents promise to improve the detection and treatment of cryptococcosis, particularly in low-income settings where the disease burden is high.

## 1. Introduction

Cryptococcosis, an invasive mycosis caused by basidiomycetous yeasts of the *Cryptococcus neoformans* or *Cryptococcus gattii* species complexes, is a cause of significant morbidity and mortality. Although *Cryptococcus* spp. are widely distributed in the environment, with over 30 species identified, only two—*C. neoformans* and *C. gattii*—cause the majority of human infection. Cryptococcosis is typically acquired via the respiratory tract through the inhalation of desiccated yeast cells or basidiospores with resultant primary lung infection [1]. 

In addition to causing localised respiratory disease, *Cryptococcus* spp. are neurotropic, with meningoencephalitis a frequent manifestation, often carrying long-term sequelae. Combined lung and central nervous system (CNS) infection is common [2]. Other sites of infection include the musculoskeletal system, skin and soft tissues, prostate, abdominal organs and eye which may occur either in combination with lung and/or CNS infection or in isolation [1]. Although *C. neoformans* and *C. gattii* infection share common clinical features, awareness of species-specific differences is important, including in the manifestations of lung disease such as pulmonary mass lesions [3,4]. Best appreciated as an opportunistic pathogen in immunocompromised populations, it is noteworthy that *Cryptococcus* spp., in particular *C. gattii*, also cause severe disease in immunocompetent hosts [5]. An appreciation of potential differences in disease presentations in these two broad host groups is required to inform best practice management of infection. 

*Cryptococcus* spp. are quintessential One Health pathogens of concern across the human, animal and environmental sectors [6]. In the veterinary space, *Cryptococcus* spp. are known to cause disease including severe lung infection in a wide range of hosts from koalas to dolphins [6], highlighting the ubiquitous nature of this opportunistic pathogen. Ongoing concerns regarding escalating antifungal resistance likely stem from environmental contamination by azoles from the agricultural sector, with substantial implications for treatment of infection in humans and animals [7].

The Australasian Society of Infectious Diseases [8] and the Infectious Diseases Society of America (IDSA) have produced guidelines [9] to assist clinicians with management of pulmonary cryptococcosis, alongside a number of reviews detailing the important features of pulmonary cryptococcosis [5,10,11]. Here, we focus on the epidemiology, clinical presentation and management of pulmonary cryptococcosis in the era of modern diagnostic methods, imaging improvements and widespread azole availability. We also highlight management priorities in special populations such as pregnant women and children, and discuss key opportunities for development in terms of both diagnosis and treatment of pulmonary cryptococcosis. 

## 2. Epidemiology and Host Risk Factors

As acquisition of cryptococcosis occurs typically via inhalation, the lung is a key target organ. Rare cases of infection have been acquired from solid organ transplantation [12], but otherwise human-to-human transmission has not been documented. Cryptococcosis manifests across the age range; although rare in children, it has been well described [13].

**Figure 1:**
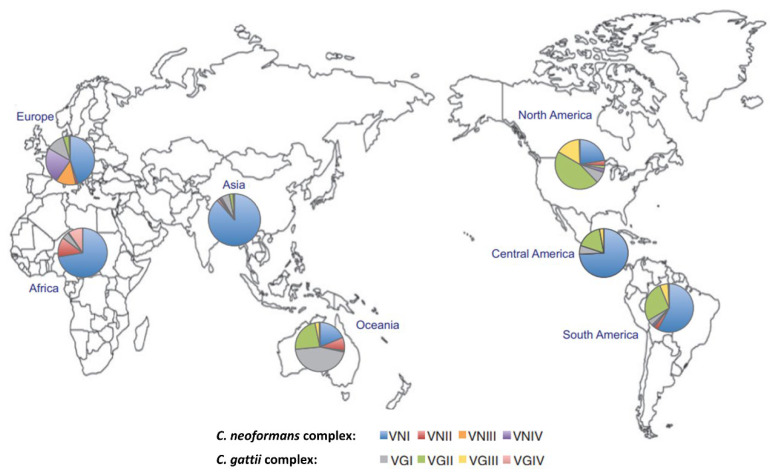
Geographical distribution of the major molecular types within the *Cryptococcus neoformans* and *Cryptococcus gattii* species complexes. Authors’ adaptation from [25].

*C. neoformans* complex is the predominant pathogen worldwide (Figure 1), with the majority of infections due to *C. neoformans* var. *grubii* (serotype A, see Table 1). Infection is often associated with circumstantial exposure to pigeon or chicken guano, although rotting vegetative matter, dairy products and soil have also been implicated [14,15]. Disease due to *C. neoformans* var. *neoformans* (serotype D) is seen mostly in Europe, accounting for approximately a third of cases. 

*C. gattii* complex forms a major aetiological component of cryptococcosis in certain regions such as Australia, Papua New Guinea, parts of the USA and in Canada. *C. gattii* is found in vegetation, such as eucalyptus and other tree species, with case clusters reported in Vancouver Island and the USA in broad proximity to these environmental reservoirs [16]. While *C. gattii* was initially thought to be geographically restricted to subtropical and tropical pockets, its distribution is now more widespread, with historical limitations attributed in part to a lack of species differentiation [17]. 

The nomenclature of *C. neoformans* and *C. gattii* subtypes has been revised in line with updated information from DNA sequencing. Additional *Cryptococcus* species names have been suggested [18] but in practice, the nomenclature *C. neoformans* or *C. gattii* complex are used in most settings [19]. Genotypes of *C. neoformans* include VNI, VNBI, VNII, VNBII, VNIII and VNIV and *C. gattii* genotypes encompass VGI, VGII, VGIIa, VGIIb, VGIIc, VGIII, VGIV, VGV and VGIV (Table 1) [18,19], with the latter two *C. gattii* genotypes, (VGV and VGVI) only recently recognised [20,21]. An alternative classification of the pathogenic *Cryptococcus* species into seven unique species within a *C. neoformans*/*C. gattii* complex has also been proposed [18] (Table 2). Worldwide distribution varies significantly by genotype (Figure 1) [18,19]. 

Hybrid AD strains of *C. neoformans* are also well recognised [22]; these are globally distributed with ancestral strains likely originating in sub-Saharan Africa [23]. However, the implications of this hybrid genotype on pathogenesis and clinical prognosis are as yet unclear. Preliminary human clinical data suggest improved prognosis in infections due to AD hybrid strains compared to serotype A and serotype D [24], although increased mortality was demonstrated in initial murine models [22]. It has been suggested that adaptive evolution of *C. neoformans* to generate such diploid hybrid variants may confer increased fitness in terms of persistence in the environment as well as in the host.

**Table 1:** Species, genotype and serotype groups of *Cryptococcus* species. Modified from [18,20,26].

Species Complex and Historical Variants	Genotype (Molecular Subtype)	Historical Serotype	Proposed Species Name [18]
*C. neoformans* complex			
*C. neoformans* var. *grubii*	VNI, VNII, VNBI, VNBII and VNIII	A	*C. neoformans*
*C. neoformans* var. *neoformans*	VNIV	D	*C. deneoformans*
*C. neoformans* hybrid *	VNIII	AD hybrid	*C. neoformans* × *C. deneoformans* hybrid
*C. gattii* complex			
*C. gattii*	VGI VGII, VGIIa, VGIIb, VGIIc VGIII VGIV VGV VGVI	B B C B and C B B	*C. gattii* *C. deuterogattii* *C. bacillisporus* *C. tetragattii* - ^†^ *C. decagattii*

* Diploid serotype AD hybrid forms of *C. neoformans* complex [22]. ^†^ Species name not yet assigned.

Immunocompromise, especially T-cell mediated deficiencies including those due to human immunodeficiency virus (HIV)/acquired immunodeficiency syndrome (AIDS), predispose to cryptococcal infection. In HIV/AIDS, infection typically manifests with CD4 T-lymphocyte counts under 100 cells/μL [26]. Other risk factors for cryptococcal disease include solid organ transplant [27], calcineurin inhibitors and other immunomodulatory agents [28], auto-antibodies against granulocyte-macrophage-colony stimulating factor (anti-GM-CSF) [29], decompensated cirrhosis [28], type 2 diabetes mellitus, malignant solid tumours, autoimmune diseases and chronic kidney disease [30]. Pulmonary disease in all these contexts is common although dissemination has been reported to be associated with pre-existing renal impairment [31]. *C. neoformans* is the predominant pathogen although there is substantial overlap in the disease spectrum between the two pathogenic species complexes [4,32].

## 3. Clinical Manifestations

**Figure 2:**
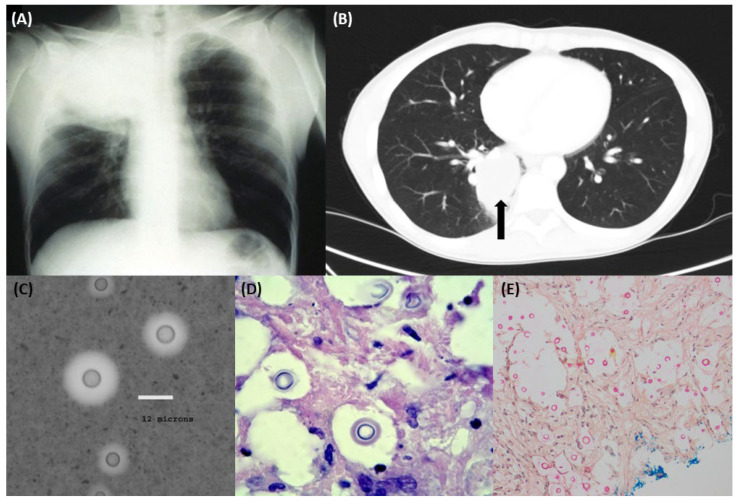
Representative (**A**) chest X-ray showing pulmonary *Cryptococcus gattii* infection with associated Pancoast syndrome (adapted from [33] by courtesy of Oxford Academic); (**B**) chest high-resolution computed tomography image of a pulmonary cryptococcoma (axial view); (**C**) India ink stain of *Cryptococcus* spp.; (**D**) periodic acid-Schiff and (**E**) mucicarmine stains of lung tissue with *Cryptococcus* spp. seen (with capsule staining bright pink in colour).

The manifestations of pulmonary cryptococcosis are protean, spanning from an isolated lung mass (ranging from a simple nodule to a large cavitatory mass) (Figure 2A,B), to multiple pulmonary nodules or a widespread disseminated interstitial infection [5]. These lung findings may occur in isolation or in association with CNS infection, particularly meningoencephalitis and disseminated disease [2]. This spectrum of clinical presentations varies depending on the immune status of the host and on the causative species, as discussed below.

Isolated cryptococcomas are a common respiratory manifestation in immunocompetent patients. In those without other comorbidities, cough (22.3%), chest pain (10.4%), sputum production (6.0%) and/or fever (23%) are the most frequent symptoms [34]. Pulmonary cryptococcosis is asymptomatic in up to 25–55% of cases [35,36] and presents as an incidental finding on chest imaging performed for another reason (as discussed below). In severe or untreated HIV/AIDS infection, fulminant disease with pulmonary infiltrates may occur [37]. Acute respiratory failure portends high mortality rates, reported at 100% in one small case series [37]. 

Of note, the clinical presentation may be indistinguishable from other causes of opportunistic pneumomia. In high tuberculosis (TB)-burden settings, the clinical picture may be indistinguishable from TB and indeed concurrent infections are well described [38]; hence, a high index of suspicion for cryptococcosis is required. In patients with HIV/AIDS, *Pneumocystis jirovecii* pneumonia (PJP) is another differential necessitating appropriate diagnostic tests. 

Of key importance, disseminated infection (especially CNS infection) must be specifically considered in all patients with pulmonary cryptococcosis to ensure treatment modality and duration is appropriate [9,39]. A recent meta-analysis of patients with HIV coinfection found that asymptomatic cryptococcal meningitis occurred in 33% of patients with cryptococcal antigenemia [40]; hence, a lumbar puncture and neuroimaging must form part of the routine diagnostics for all patients with cryptococcal infection. Cryptococcal meningitis typically presents with a subacute or chronic headache with or without symptoms of raised intracranial pressure (nausea, vomiting, photophobia and/or blurred vision). Neck stiffness, reduced or fluctuating level of consciousness or confusion may also be present. Infection at other sites such as skin, bone and joints, prostate and eyes may be revealed by an appropriate symptom complex or imaging findings. Non-specific symptoms such as fever and malaise are not uncommon. 

### Comparison of C. gattii with C. neoformans Infection

Broadly, the clinical spectrum of *C. gattii* infection overlaps with that of *C. neoformans* and the same diagnostic modalities are employed. In many geographic regions, it is recommended that clinical isolates be routinely differentiated into *C. neoformans* complex and *C. gattii* complex for clinical and epidemiological purposes [8]. 

Similar to *C. neoformans*, pulmonary involvement is seen in about two thirds of *C. gattii* infections, mostly with concurrent CNS disease [41]. The pulmonary lesions in *C. gattii* may be very large (over 5–10 cm), with Pancoast syndrome a well-described complication [33] (Figure 2A). In meningoencephalitis, complications are common including raised intracranial pressure and hydrocephalus, often with severe neurological sequelae [42,43,44]. Brain cryptococcomas too are more frequent with *C. gattii* [45], although the attribution of this feature to pathogen-specific versus host factors is not fully elucidated. 

Some distinctions between clinical features of *C. neoformans* and *C. gattii* infection may be explained by differences in host population [46]. Overall, *C. gattii* causes disease more frequently in persons with apparently normal immune systems, in contrast to *C. neoformans*, which commonly affects individuals with cell-mediated immune deficiencies [4,32]. As above, *C. gattii* infection is associated with a higher incidence of large cryptococcomas which can significantly influence management pathways [47,48]. Interestingly, the presence of autoantibodies against granulocyte-macrophage-colony stimulating factor (anti-GM-CSF) has specifically been associated with cryptococcal meningitis caused by *C. gattii* but not *C. neoformans*, though the mechanism is unclear [29,41,49,50].

## 4. Diagnosis

Since symptoms are often non-specific, diagnosis of cryptococcosis is often delayed with patients either treated for bacterial pneumonia or mistakenly diagnosed with lung carcinoma. Radiological findings are pivotal in understanding the nature and extent of disease. However, the mainstay of diagnosis relies on histopathology, culture-based methods and adjunctive cryptococcal antigen (CrAg) testing which confers high specificity and at least moderate sensitivity in pulmonary disease [51,52,53]. Detection of *Cryptococcus*-specific nucleic acid sequences by molecular methods has a role in certain clinical contexts, although this is generally not performed routinely. Given the ubiquitous nature of *Cryptococcus* in the environment and high seroprevalence in some populations [54], antibody-based serological methods have limited use in diagnosis, though provide valuable information to inform epidemiological surveys. 

Collection of appropriate specimens is critical to optimising diagnostic yield. Serum should be collected for detection of CrAg. Lung tissue samples taken from the site of disease via percutaneous or trans-bronchial biopsy are the optimal specimen types, particularly since many cryptococcal lung lesions are located peripherally [55]. These specimens should not be placed in formalin prior to culture. In the absence of tissue samples, bronchoalveolar lavage fluid (BALF) specimens also afford high-quality lower airway specimens, particularly for more diffuse disease such as in immunocompromised patients. 

Sampling of cerebrospinal fluid (CSF) via a lumbar puncture and appropriate brain imaging must be performed to exclude CNS infection in all patients with pulmonary cryptococcosis [9,39], given the disease often manifests concurrently at these two sites. Increased intracranial pressure is common; hence, CSF opening pressure should always be recorded.

Classification of pulmonary cryptococcosis into mild-to-moderate or severe disease enables appropriate selection of a treatment regimen. Mild-to-moderate pulmonary cryptococcosis is defined by the absence of diffuse pulmonary infiltrates and lack of dissemination to other anatomical sites, while severe disease is defined as the presence of multiple pulmonary cryptococcomas, diffuse pulmonary infiltrates and/or dissemination to other sites including the CNS [9,56].

### 4.1. Radiology

Chest imaging, using a plain radiograph (Figure 2A) or high-resolution computed tomograph (HRCT) (Figure 2B), forms a cornerstone of the diagnostic toolkit for pulmonary cryptococcosis. Patients may have single or multiple parenchymal nodules, which are often subpleural [34], and cavitation may be seen particularly in immunocompromised patients [57]. 

In asymptomatic patients, an abnormality on chest radiograph is often the first indication of the infection, attesting to the key role of radiology in diagnosis of pulmonary cryptococcosis [57]. It is not unusual for such cases to be provisionally diagnosed as pulmonary malignancy, as these lesions present similar radiology. The finding of encapsulated yeasts on microscopy, histopathology or culture of a biopsy or BALF specimen is frequently an unexpected finding. Such sampling, however, facilitates accurate diagnosis and exclusion of alternative differentials and enables directed testing to guide choice of an appropriate antifungal regimen. Imaging of other affected sites should be performed as clinically indicated. 

### 4.2. Cryptococcal Antigen Testing

Cryptococcal antigen (CrAg) testing may be conducted using lateral flow immunochromatography or latex agglutination approaches. The lateral flow CrAg assay, now the most widely used, detects the presence of capsular glucuronoxylomannan present in both *C. neoformans* and *C. gattii* [58]. Its low cost and ease of use has led to widespread application, and in lower income settings it has revolutionised the ability of clinicians to reach accurate and timely diagnoses [51]. The assay has high sensitivity and specificity (>95% and 100%, respectively, in CSF and sera of patients with HIV) [51,52,53]. In patients with pulmonary disease and without HIV, sensitivity and specificity of serum CrAg remain high (97% and 95% respectively in one study) [59]. The possibility for a prozone effect can be offset by running the assay at a 5- or 10-fold dilution alongside the neat specimen [60], and a positive result should always be titred to endpoint using serial dilutions. 

In patients with pulmonary cryptococcosis, median serum CrAg levels of 1:16 are seen in immunocompetent hosts, or 1:32 in immunocompromised persons [34]. Significantly higher CrAg titres have been demonstrated in patients with meningitis compared to those without, and this is consistent across immunocompetent [61] and immunocompromised patients [62]. However, clinicians should be aware that a negative CrAg result may occur in immunosuppressed patients, particularly in the context of an isolated pulmonary lesion [63]. Due to variable changes in CrAg levels with and without treatment, this marker should not be used as an indicator of response to treatment, though it does generally correlate with organism burden at time of diagnosis [11].

### 4.3. Microscopy

As encapsulated yeasts, *Cryptococcus* spp. are readily recognised on a wet preparation by the presence of a thick gelatinous capsule (though some strains do fail to produce a capsule on subculture) [64]. Distinctly, India ink staining gives the characteristic appearance of a refractile unstained capsule against the darkly staining background but this is only performed on CSF samples (Figure 2C) [65]. On Gram stain, *Cryptococcus* spp. appear as narrow-based budding cells with Gram positive inclusions, sometimes visible against a non-staining or poorly staining capsule [65].

### 4.4. Histopathology

Histopathological assessment reveals pathognomonic findings in pulmonary cryptococcosis. Macroscopically, excised cryptococcomas appear as one or more firm nodules with a white or cream cut surface which may be mucoid [66]. On microscopy, Grocott-Gomori methenamine silver (GMS), periodic acid Schiff (PAS) (Figure 2D) and haematoxylin and eosin (H&E) stains frequently reveal yeast cells within the necrotic centre of the nodule, though these may be sparse, and there are often associated signs of tissue invasion. The necrotic zone is typically delineated by palisading macrophages, surrounded by a mixed inflammatory infiltrate of lymphocytes, plasma cells and fibroblasts [66]. Foamy histiocytes may also be present. The narrow budding characteristic of *Cryptococcus* spp. as well as the absence of pseudohyphae provide additional morphological hallmarks to differentiate them from other yeasts such as Candida. More *Cryptococcus*-specific stains such as mucicarmine (which stains the polysaccharide capsule) (Figure 2E) or Fontana-Masson stains (which highlight the melanin pigment) may also be used [67].

### 4.5. Culture and Susceptibility Testing

Lung tissue or BALF specimens provide optimal sensitivity and specificity for culture of *Cryptococcus* spp. in lung infection. Blood cultures may also yield *Cryptococcus* spp. although very uncommonly in isolated lung disease. Induced or expectorated sputa are not generally recommended as specimen types when Cryptococcus is suspected, due to contamination with upper respiratory commensal flora. Irrespective, the finding of *C. neoformans* or *C. gattii* in any sputum specimen should be reported to the clinician for further assessment of the presence of cryptococcal disease with a chest radiograph and lower respiratory sampling. 

*Cryptococcus* spp. grow as glistening mucoid colonies on routine fungal culture media (such as Sabouraud’s dextrose agar) under aerobic conditions with an optimal temperature of 30 °C. Unlike other *Cryptococcus* spp., most *C. neoformans* and *C. gattii* isolates will tolerate temperatures up to 37 °C which is a key diagnostic feature [68]. Growth is typically seen within 7 days although incubation up to 14 days is recommended. The use of differential media such as bird seed agar may be useful diagnostically, with *Cryptococcus* spp. growing as characteristic brown colonies due to formation of melanin from the biphenol components of the media [69]. The ability of *Cryptococcus* spp. to hydrolyse urea is another distinguishing biochemical feature. 

Historically, serotyping of *Cryptococcus* spp. was performed using capsular agglutination reactions to the four historical serotypes–A, B, C and D (Table 1) but this is now rarely performed. In most laboratories, matrix assisted laser desorption ionisation time of flight mass spectrometry (MALDI-ToF MS) based techniques using either the Bruker Biotyper (Bruker Daltoniks, GmbH, Bremen, Germany) or Vitek MS (BioMerieux, Marcy-l’etoile, France) systems provide reliable species and subspecies level identification [70,71,72,73]. Molecular identification may also be performed using sequencing of the internal transcribed spacer (ITS) region, D1/D2 regions or cryptococcal *cytB* gene for identification of unresolved isolates, which provides sufficient detail for sub-classification [74].

Routine susceptibility testing is not recommended in cryptococcosis due to insufficient evidence to support correlation between MICs and clinical outcomes [8]. Its main role is in patients failing to respond to first line therapy and in MIC surveillance in reference laboratories to monitor epidemiological trends over time [75,76]. 

If indicated, susceptibility testing is performed using a reference broth microdilution method (i.e., Clinical & Laboratory Standards Institute (CLSI) [77]/European Committee on Antimicrobial Susceptibility Testing (EUCAST) [78]) or a commercial equivalent such as the YeastONE^TM^ Sensititre assay (ThermoFisher Scientific, Waltham, MA, USA) or the VITEK 2 system (bioMérieux, Inc., Hazelwood, MO, USA) [79]. For *C. neoformans*, there are breakpoints only for amphotericin B using EUCAST methodologies (susceptible isolates corresponding to a MIC ≤ 1 mg/L) [80]; no clinical breakpoints are available for *C. gattii*. CLSI methods do not have clinical breakpoints for *C. neoformans* or *C. gattii*, although epidemiological cutoff values (ECVs) have been determined [81].

*Cryptococcus* spp. have largely retained low minimum inhibitory concentrations (MICs) to amphotericin B and flucytosine, but are uniformly resistant to echinocandins (though the mechanism is not well established). Concerningly, a trend of increasing fluconazole MICs has been described [7]. Moreover, one systematic review found that while approximately 12% of isolates were non-wildtype with respect to fluconazole at baseline, relapsed infections were associated with increased rates of elevated MICs (24%), although the included studies used differing fluconazole MIC thresholds (ranging from ≥16 to ≥64 μg/mL) [82]. Amphotericin B resistance has been reported but remains rare [83]. 

Similar to *C. neoformans*, several studies have documented comparatively low MICs of standard antifungals against *C. gattii*, including amphotericin B and 5-flucytosine. Although some MICs appeared to be relatively higher for *C. gattii* than for *C. neoformans*, the clinical significance of this is unclear [84,85,86,87,88]. 

### 4.6. Nucleic Acid Amplification Testing

Nucleic acid amplification testing (NAAT) has previously been a minor contributor to the diagnostic toolkit for cryptococcosis but now may be coming into its own. Most commercial platforms, including multiplexed assays such as the AusDiagnostics atypical pneumonia or CSF panels (AusDiagnostics, Mascot, Australia) and BioFire meningitis/encephalitis (ME) panel (bioMérieux, Marcy l’Etoile, France) target the ITS region of *Cryptococcus* spp. but do not differentiate between *C. neoformans* and *C. gattii* [89,90,91]; they are validated for use only for specific specimen types. The sensitivity of the BioFire ME assay (bioMerieux) for detection of *Cryptococcus* spp. is reported at 84% compared to CrAg [91], so should not be used in isolation as a rule-out test. 

Panfungal PCR assays accompanied by sequencing of the ITS amplicon provide detailed genotypic information which enables accurate speciation, but lengthy turnaround times can be problematic for achieving a prompt diagnosis in the acute phase, and the assay performs most optimally on tissue specimens where yeasts have been visualised on microscopy [92]. New molecular assays, targeting the *cytb* gene have enabled identification of *C. neoformans* and *C. gattii* with 96% sensitivity and 100% specificity [93]. Such assays, yet to enter mainstream use, will form a powerful diagnostic tool enabling speciation in cases of pulmonary cryptococcosis. 

## 5. Treatment of Pulmonary Cryptococcosis

Choice and duration of treatment for pulmonary cryptococcosis is dependent on host factors, primarily immune status and the severity and extent of disease [8,9,56,94]. Clinical practice guidelines for management of pulmonary cryptococcosis are available from the Infectious Diseases Society of America (IDSA), American Society of Transplantation (AST) and Australasian Antifungal Guidelines Steering Committee [8,9,95]. Current recommendations for management of *C. gattii* infections are based largely on extrapolation from clinical trials of *C. neoformans* and on case series, individual case reports and expert opinion. 

Surgical resection is recommended in patients with large, surgically accessible pulmonary cryptococcomas [47,48] or those with persistent symptoms and active or progressive radiological changes despite antifungal therapy [9,39,56,96]. Infectious diseases consultation has been shown to improve outcomes, particularly in the setting of severe disease [97]. 

In immunocompetent individuals there have been multiple reports of spontaneous resolution of pulmonary cryptococcal lesions, particularly in the asymptomatic population [98]. However, due to the risk of disseminated disease and its associated morbidity, most guidelines and expert bodies recommend treatment, even for asymptomatic or mild-to-moderate pulmonary cryptococcosis (Table 2) [9,56,99,100]. Treatment is generally given for the prescribed period, dependent on clinical response; a persistently positive serum CrAg or radiological findings at the conclusion of the treatment course are not indications for prolongation of therapy if the patient is otherwise well [9,99]. Alternative therapy may be required if fluconazole is contraindicated or unavailable. Options include itraconazole, voriconazole or posaconazole, though experience is limited to case series only [9,101,102,103,104]. Results from isavuconazole use are mixed amongst small case series, with both successful and unsuccessful outcomes reported [105,106]. Data from large patient cohorts, particularly in the form of prospective randomised trials, are awaited to further delineate the utility of this newer agent in management of cryptococcal disease.

Severe pulmonary disease—defined as the presence of multiple pulmonary cryptococcomas, diffuse pulmonary infiltrates and/or dissemination to other sites including the CNS—should be treated similarly to cryptococcal meningitis. An induction phase with combination intravenous amphotericin B and 5-flucytosine for two to four weeks is followed by consolidation and maintenance phases with oral fluconazole for at least one year (Table 2) [5,9,39,56]. Single high-dose liposomal amphotericin combined with 14 days of oral flucytosine and fluconazole is now recommended by the World Health Organization as induction therapy for cryptococcal meningitis in patients with HIV infection [107], on the basis of recent randomised controlled trial data [108]. Outcome data for similar regimens in pulmonary disease are awaited. It is possible, but has not yet been tested, that short course parenteral regimens will enter mainstream practice for cryptococcal disease in other body sites. 

**Table 2:** Treatment and dosage guidance for management of *Cryptococcus neoformans* and *Cryptococcus gattii* pulmonary infections. Modified from the Infectious Diseases Society of America (IDSA), American Society of Transplantation (AST), Australasian Antifungal Guidelines Steering Committee and the American Thoracic Society guidelines [8,9,39,95].

Host Risk Group/Severity of Disease	First Line Antifungal Therapy	Alternative Antifungal Regimen	Duration of Therapy/Comments
Immunocompetent - Mild-to-moderate	Fluconazole 400–800 mg orally, daily	Itraconazole (loading doses of 200 mg orally three times daily for three days, then 200 mg orally twice daily) Voriconazole (loading doses of 6 mg/kg intravenously twice daily or 400 mg orally twice daily on the first day, then 200 mg orally twice daily) Posaconazole delayed-release tablets (loading doses of 300 mg orally twice daily on the first day, then 300 mg orally once daily)	6 to 12 months
- Severe	**Induction**: Liposomal amphotericin 3 mg/kg/day intravenously (or conventional amphotericin 0.7–1.0 mg/kg/day) plus flucytosine 100 mg/kg/day orally **Consolidation**: Fluconazole 400–800 mg orally, daily **Maintenance**: Fluconazole 200-400 mg orally, daily		**Induction**: 2–4 weeks **Consolidation**: 8 weeks **Maintenance**: 12 months
Immunocompromised—HIV and non-HIV - Mild-to-moderate	Fluconazole 400 mg orally, daily	Itraconazole (loading doses of 200 mg orally three times daily for three days, then 200 mg orally twice daily) Voriconazole (loading doses of 6 mg/kg intravenously twice daily or 400 mg orally twice daily on the first day, then 200 mg orally twice daily) Posaconazole delayed-release tablets (loading doses of 300 mg orally twice daily on the first day, then 300 mg orally once daily)	6 to 12 months In patients with HIV coinfection, suppressive therapy (fluconazole 200 mg orally, daily) should be given after the acute treatment course. This may be ceased after 12 months if CD4 count > 100 cells/μL, HIV viral load is undetectable and cryptococcal antigen titre is stable at <1:512.
- Severe	Treat as for immunocompetent, severe disease		
Pregnant women - Mild-to-moderate	“Watch and wait”, close clinical monitoring	Seek expert opinion Consider amphotericin in 1st trimester; fluconazole 400 mg orally, daily in 2nd and 3rd trimester if required	Recommended to defer treatment until after delivery unless severe disease
- Severe	Treat as for immunocompetent, severe disease with expert input		
Children - Mild-to-moderate	Fluconazole 6–12 mg/kg orally, daily	Treat as for children, severe disease	6 to 12 months
- Severe	**Induction**: Amphotericin B 1 mg/kg per day intravenously plus flucytosine 100 mg/kg per day orally (in 4 divided doses) **Consolidation**: fluconazole 10–12 mg/kg per day orally **Maintenance**: fluconazole 6–12 mg/kg per day orally	Liposomal Amphotericin B 5 mg/kg per day intravenously (or Amphotericin B lipid complex 5 mg/kg per day intravenously)	**Induction**: 2 weeks **Consolidation**: 8 weeks **Maintenance**: 6 to 12 months

The role of corticosteroids in optimising outcomes for cryptococcal disease is unclear, but generally these agents are not recommended. In patients with acute respiratory distress syndrome and no CNS disease, corticosteroids may be considered [9]. In cryptococcal disease of the CNS, however, steroids are not recommended since randomised trial data (in HIV positive individuals) demonstrated no reduction in mortality with dexamethasone use, but a significant increase in adverse effects and morbidity [109]. As this trial was terminated early, there were too few patients with CNS cryptococcomas to comment on the utility of steroids in this predefined subgroup. Interesting, an earlier retrospective study had found a marked improvement in clinical outcome in patients who received adjunctive steroids for meningitis due to *C. gattii* [110]. Consultation with an infectious diseases expert is recommended for the management of patients with CNS cryptococcomas.

Management of *C. gattii* infections may require modification of the aforementioned recommendations. In particular, a longer duration of induction as well as total duration of therapy may be indicated [48], largely due to the association with larger cryptococcomas and more severe disease. Furthermore, given the relative frequency of large cryptococcomas in *C. gattii* infections, surgical excision is often an important aspect of achieving an effective cure, in conjunction with antifungal therapy [47,48]. 

New agents under investigation for management of pulmonary cryptococcosis include the novel antifungal agents APX001A (Manogepix), VT-1129 and T-2307, which have demonstrated in vivo and in vitro activity against *Cryptococcus* spp., and detailed Phase II and Phase III clinical data are awaited [111,112,113,114,115,116]. The use of sertraline as an adjunct to standard therapy in meningitis and cryptococcal antigenaemia has been investigated. Although early in vitro and in vivo (murine) data showed promise [117], human trials did not show any additional benefit compared to conventional therapy, with one study ceasing due to severe side effects [118,119].

### 5.1. Special Populations

#### 5.1.1. HIV

Severe cryptococcal disease in patients with HIV infection should be managed as per recommendations for immunocompetent hosts. It is recommended that HIV-infected individuals with asymptomatic or mild-to-moderate pulmonary disease receive fluconazole therapy [9,39,120], although some groups advocate for use of “severe disease” treatment protocols even in mild disease (Table 2) due to the high risk of dissemination in patients with HIV [56]. Suppressive therapy with oral fluconazole is recommended following the treatment phase. In a subset of patients receiving antiretroviral therapy and with a CD4 count over 100 cells/μL, undetectable viral load and a stable cryptococcal antigen titre ≤1:512, this could be ceased after 12 months [9,39,120,121,122,123]. There is a paucity of data on the optimal timing of commencement of antiretrovirals in isolated pulmonary cryptococcosis, with initiation suggested two weeks after the commencement of antifungals to minimise the risk of immune reconstitution inflammatory syndrome (IRIS) especially with cryptococcal CNS disease [120].

#### 5.1.2. Immunocompromised Non-HIV

Immunocompromised individuals without HIV with severe cryptococcosis should be managed with induction, consolidation and maintenance therapy phases, as recommended for other patient groups. Patients with asymptomatic or mild-to-moderate pulmonary disease should receive standard fluconazole therapy as outlined above [9,39,95]. As with HIV-infected individuals, some groups advocate for “severe disease” treatment protocols in mild-to-moderate disease (Table 2) [56]. Ongoing suppressive therapy is generally not required.

Drug–drug interactions can be a significant challenge in this context. Careful QTc and therapeutic drug monitoring should be performed particularly when calcineurin inhibitors are co-administered with fluconazole due to known drug–drug interactions [124]. Alternative therapies include triazole antifungals; however, these agents are more toxic and also have a high potential to interact with immunosuppressive agents. Hence, these second line therapies are recommended only when fluconazole is unavailable, contraindicated or where decreased susceptibility has been demonstrated [106,124,125,126].

#### 5.1.3. Pregnancy

Data on the optimal management of pregnant women with pulmonary cryptococcosis are limited to case reports. Vertical transmission is rare, with isolated cases reported in the context of severe disseminated maternal disease with or without HIV coinfection [127,128]. For pregnant women with asymptomatic or mild-to-moderate disease, treatment should be delayed until after delivery when it is safe to do so, and always with close monitoring for evidence of disease progression or dissemination [129,130,131,132]. Where treatment is necessary in the first trimester, amphotericin B monotherapy is suggested due to the risk of teratogenicity associated with fluconazole [9]. Treatment considerations in the second or third trimester should be balanced with the risk to the fetus. Oral fluconazole is the treatment of choice post-partum [5,9]. Breastfeeding is generally not recommended as fluconazole passes into the breastmilk at concentrations similar to plasma levels. Close monitoring after treatment initiation is critical since IRIS in the post-partum period has been reported [132].

#### 5.1.4. Children

Treatment of cryptococcosis in children is extrapolated from adult data as there are no randomised controlled trial data. While pulmonary cryptococcosis is rare in children, a high proportion of these present with disseminated disease [13,133]. For children with mild-to-moderate pulmonary cryptococcosis, weight-adjusted oral fluconazole for 6 to 12 months is recommended (Table 2) [9,13,56,134]. Amphotericin B with or without 5-flucytosine has been a successful alternative in some cases [135]. Treatment of severe disease is guided by adult recommendations with weight-adjusted dosing of agents in the induction, consolidation and maintenance phases (Table 2).

## 6. Prognosis

Mortality rates in pulmonary cryptococcosis are improving over time, but still vary widely based on immune status and comorbidities of the host as well as the presence or absence of concurrent meningitis or disseminated cryptococcosis [136]. Accurate prognostic estimates are also hindered by likely underdiagnosis of asymptomatic and/or mild cases which would carry a more favourable prognosis. Mortality in pulmonary cryptococcosis was reported at up to 55% in non-HIV patients [137], and 74% in patients with HIV in the 1990-2000s [138], though it was notable that all of the fatalities in these studies involved disseminated disease. Recent reports of 90-day mortality in patients with underlying cirrhosis (57%) [139] or following solid organ transplants (14%) [140] vary widely. Broader cross-sectional studies incorporating both immunocompetent and immunocompromised patients have reported an overall 70% treatment success rate [141]. 

## 7. Conclusions

Pulmonary cryptococcosis remains a concerning invasive mycosis, associated with substantial morbidity and mortality. Clinicians must be attuned to this condition as a differential diagnosis in patients with non-remitting respiratory symptoms, chest pain, fever and/or systemic features, or in those with the radiological finding of a single or multiple chest lesions. An accurate diagnosis requires close coordination between the treating physician, microbiologist, histopathologist and radiologist to collate the information gleaned from culture, microscopy, histology and imaging findings. Missed or late diagnoses remain commonplace, particularly in asymptomatic cases or those with an indolent course. 

Ongoing research into improved diagnostic tools and algorithms as well as trials of novel targeted antifungals, adjunctive agents and alternative treatment regimens are eagerly anticipated. More sophisticated diagnostic approaches are in development to enable timely and accurate diagnosis of *Cryptococcus* isolates to the species level [93]. The use of such NAAT assays is not yet widespread but has potential to form a powerful adjunct to existing culture, antigen and microscopy methods. Additionally, promising emergent treatments may provide improved pathways to timely and effective management of pulmonary cryptococcosis.

The ‘holy grail’ in management of pulmonary cryptococcosis would be a low-cost all-oral regimen to enable greater accessibility across resource limited settings as well as high-income countries. Such developments will be critical to ensure the optimisation of tolerability, quality of life and short- and long-term outcomes for the multitude of patients affected by pulmonary cryptococcosis worldwide.

## Abbreviations

AIDS	acquired immunodeficiency syndrome
AST	American Society of Transplantation
BALF	bronchoalveolar lavage fluid
CLSI	Clinical & Laboratory Standards Institute
CNS	central nervous system
CSF	cerebrospinal fluid
CrAg	Cryptococcal antigen
DNA	deoxyribonucleic acid
ECV	epidemiological cutoff value
EUCAST	European Committee on Antimicrobial Susceptibility Testing
GM-CSF	granulocyte-macrophage-colony stimulating factor
HIV	human immunodeficiency virus
IDSA	Infectious Diseases Society of America
IRIS	immune reconstitution inflammatory syndrome
MALDI-TOF	matrix-assisted laser desorption ionisation-time of flight
MIC	minimum inhibitory concentration
NAAT	nucleic acid amplification testing
PJP	Pneumocystis jirovecii pneumonia
TB	tuberculosis
